# Pancreatic panniculitis following endoscopic retrograde cholangiopancreatography (ERCP): a case report^؆^

**DOI:** 10.1016/j.abd.2025.501211

**Published:** 2025-10-27

**Authors:** Rebecca Perez de Amorim, Laís Maria Bellaver de Almeida, Hélio Amante Miot, Juliano Vilaverde Schmitt

**Affiliations:** Department of Infectology, Dermatology, Imaging Diagnosis and Radiotherapy, Faculty of Medicine, Universidade Estadual Paulista, Botucatu, SP, Brazil

Dear Editor,

Pancreatic panniculitis is a rare cutaneous manifestation that occurs in 0.3%–3% of patients with pancreatic disease. It is postulated that damage to the pancreas causes hematogenous release of digestive enzymes, which increases the permeability of the microcirculation within the lymphatic vessels and leads to fat saponification with necrosis of the subcutaneous tissue. The most common underlying etiology is acute pancreatitis (88% of cases), while pancreatic neoplasms account for approximately 12%.^1^, ^2^, ^3^, ^4^

This case report describes a case of pancreatic panniculitis that occurred after a cholangiography examination.

A 76-year-old caucasian woman with hypertension was admitted to a referral hospital for gastroenterology care due to abdominal pain, loss of appetite, nausea, vomiting, and jaundice for three days. Laboratory tests on admission showed anemia (hemoglobin 10.2 g/dL), leukocytosis with neutrophilia (11,930 mm^3^), eosinophilia (450 mm^3^), elevated transaminases (AST 85 µ/L [normal 14–36 µ/L], ALT 331 µ/L [normal < 35 µ/L]), alkaline phosphatase (180 µ/L, normal 36–126 µ/L), gamma-GT (285 µ/L, normal 12–43 µ/L), total bilirubin (7.10 mg/dL, normal 0.2–1.3 mg/dL), and direct bilirubin (3.5 mg/dL, normal 0–0.3 mg/dL), with no change in amylase or lipase.

An abdominal CT scan was performed, which identified dilation of the intra- and extrahepatic bile ducts, with obstruction in the distal portion of the common bile duct at the level of the duodenal papilla. Subsequently, endoscopic retrograde cholangiopancreatography (ERCP) was performed, combined with sphincterotomy and insertion of a plastic biliary stent. Since no stones were identified in the common bile duct, papillitis, pancreatic head tumor, or distal cholangiocarcinoma were hypothesized as the cause of the obstruction.

Five days after the ERCP, the dermatology team was contacted due to the appearance, post-procedure, of nodular, painful, erythematous lesions located on the upper and lower limbs (Fig. 1, Fig. 2), which had worsened significantly over the previous 24 hours. A deep skin biopsy revealed spontaneous drainage of yellowish-brown material from the site. The secretion was sent for culture, which was negative. New laboratory tests revealed elevated amylase (123 µ/L, [normal 30-110 µ/L]) and lipase (618 µ/L, [normal 36-126 µ/L]). Histopathological examination revealed acute suppurative panniculitis with lymphocyte and neutrophil infiltration, steatonecrosis with foci of incipient calcification, and ghost cells (Fig. 3).

**Fig. 1:**
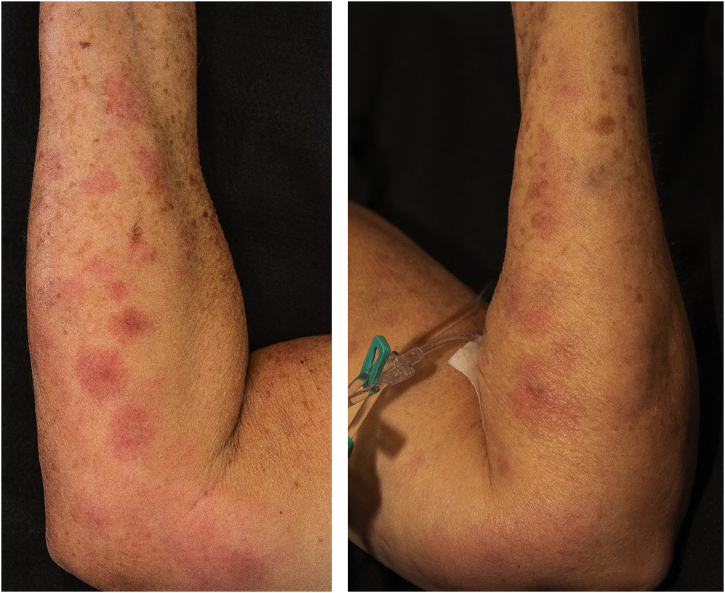
Erythematous and painful nodules affecting the entire length of the upper limbs.

**Fig. 2:**
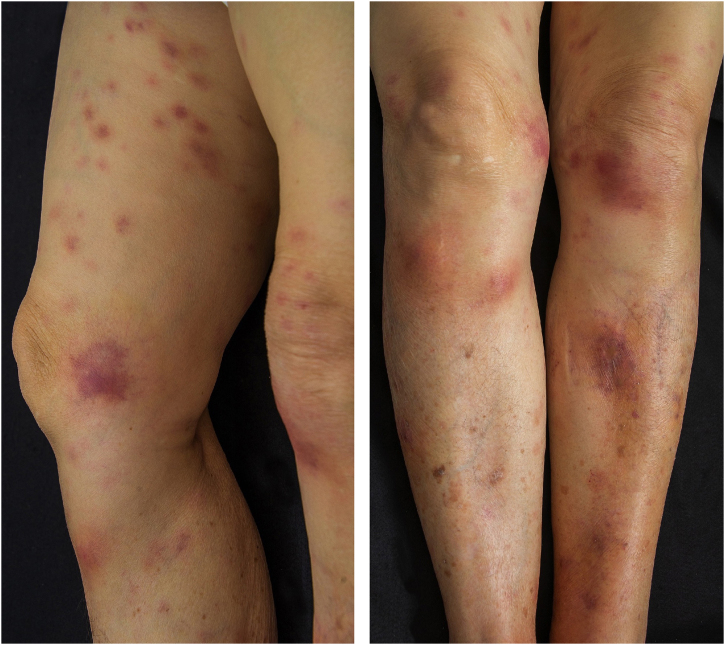
Erythematous and painful nodules affecting the entire length of the lower limbs.

**Fig. 3:**
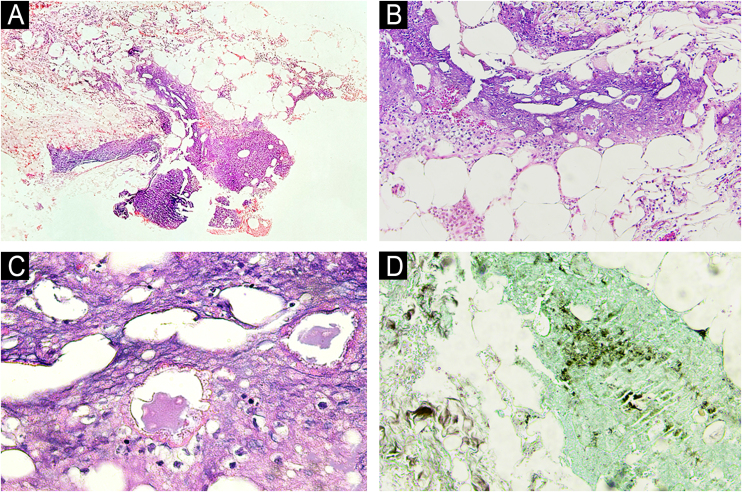
Histopathological examination of pancreatic panniculitis. (A) Acute suppurative panniculitis with lobular predominance (Hematoxylin & eosin, ×100). (B) Lymphocyte and neutrophil inflammatory infiltrate (Hematoxylin & eosin, ×200). (C) Steatonecrosis with “ghost cells” (Hematoxylin & eosin, ×400). (D) Foci of calcification amid steatonecrosis (von Kossa, ×400).

Clinical, laboratory, and histopathological findings indicated the diagnosis of pancreatic panniculitis. The treating team was advised regarding the skin condition, which should be managed symptomatically until the underlying disease is resolved, with complete spontaneous resolution within 45 days. The patient has been followed for two years with a probable diagnosis of distal cholangiocarcinoma. She has been asymptomatic since the biliary stent implantation, and surgical treatment is contraindicated due to her advanced age and comorbidities.

The characteristic lesions of pancreatic panniculitis are erythematous and painful nodules, which appear suddenly, located primarily on the lower limbs but also on the buttocks, trunk, and upper limbs. The nodules may fluctuate, ulcerate, and drain a yellow-brown oily secretion. In 40% of cases, skin lesions may precede abdominal symptoms.^1^, ^4^, ^5^

Among the differential diagnoses, it is worth mentioning: erythema nodosum, nodular vasculitis, alpha-1 antitrypsin deficiency, lupus panniculitis, subcutaneous T-cell panniculitis-like lymphoma, Bazin's indurated erythema, and other infectious panniculitis.^1^, ^2^, ^3^

Histopathological examination is classically marked by focal subcutaneous steatonecrosis and the presence of pathognomonic "ghost cells" (anucleated necrotic adipocytes with thick, obscure walls, containing fine, basophilic granular intracytoplasmic material).^1^, ^2^, ^4^, ^5^

Treatment is supportive, with resolution observed after treatment of the underlying pancreatic process. Analgesic medications such as nonsteroidal anti-inflammatory drugs, corticosteroids, and immunosuppressants are frequently used but are often ineffective.^1^, ^2^, ^4^

Acute pancreatitis after ERCP is a complication that occurs in up to 3%–10% of cases, depending on patient risk factors and the complexity of the procedure. It is characterized by acute inflammation of the pancreas associated with mechanical manipulation of the pancreatic duct, contrast injection, or edema caused by local trauma.^6^ The onset of pancreatic panniculitis after ERCP is uncommon but may precede abdominal symptoms.^7^, ^8^, ^9^, ^10^

In conclusion, this report describes a case of pancreatic panniculitis as a complication of ERCP, without prior pancreatic disease. Dermatologists should be alert to the diagnosis of this complication after biliary tract procedures.

## ORCID ID

Laís Maria Bellaver de Almeida: 0000-0002-1695-5210

Juliano Vilaverde Schmitt: 0000-0002-7975-2429

## Financial support

None declared.

## Authors’ contributions

Rebecca Perez de Amorim: Design and planning of the study; drafting and editing of the manuscript; collection, analysis, and interpretation of data; intellectual participation in the propaedeutic and/or therapeutic conduct of the studied cases; critical review of the literature; critical review of the manuscript; approval of the final version of the manuscript.

Laís Maria Bellaver de Almeida: Design and planning of the study; collection, analysis, and interpretation of data; critical review of the literature; critical review of the manuscript.

Helio Amante Miot: Critical review of the literature; critical review of the manuscript; approval of the final version of the manuscript.

Juliano Vilaverde Schmitt: Design and planning of the study; drafting and editing of the manuscript; intellectual participation in the propaedeutic and/or therapeutic conduct of the studied cases; critical review of the literature; critical review of the manuscript; approval of the final version of the manuscript.

## Research data availability

Does not apply.

## Conflicts of interest

None declared.
